# Acute Promyelocytic Leukemia: A Summary

**Published:** 2018-03-01

**Authors:** Meaghan M. Ryan

**Affiliations:** Washington University School of Medicine, Saint Louis, Missouri

## Abstract

Acute promyelocytic leukemia is a distinct subtype of acute myeloid leukemia. The disease is characterized by a chromosomal abnormality involving translocation between chromosomes 15 and 17. Current therapy has advanced to include agents such as all-*trans* retinoic acid and arsenic trioxide, which have improved remission and survival rates. Induction, consolidation, and maintenance regimens have now been studied and are outlined. As patients affected can develop severe bleeding complications, rapid diagnosis and initiation of appropriate treatment are vital. During treatment, unique complications such as disseminated intravascular coagulation and differentiation syndrome can occur. Prompt recognition of complications is imperative.

Acute promyelocytic leukemia (APL) is a rare subtype of acute myeloid leukemia, which historically had been considered one of the most fatal types of this disease process. With advances in treatment regimens, namely the introduction of all-*trans* retinoic acid (ATRA), outcomes have drastically improved, with complete remission (CR) rates approaching 100% in ATRA-based regimens and event-free survival rates recorded as more than 90%. Furthermore, 2-year overall survival probability and disease-free survival rates exceed 90% with these regimens (Lo-Coco et al., 2013). Regimens have been altered in recent years with the addition of arsenic trioxide (ATO) and anthracyclines, which have further improved outcomes. Due to a variety of schema used in clinical trials and the relatively small percentage of leukemia cases that APL patients comprise, determining a plan of action can be challenging for the oncology team. Furthermore, potentially devastating complications during induction therapy can occur, of which proper recognition and management can be life-saving. Of these complications, bleeding and differentiation syndrome are the most threatening. Advanced practitioners often play an integral role in acute leukemia patients’ hospitalizations and, in turn, often are among the first to recognize clinical changes. Close monitoring and prompt response to clinical changes lead to improved outcomes for this subset of acute leukemia patients.

## HISTORY

Acute promyelocytic leukemia was first designated as a distinct entity in 1957 (Coombs, Tavakkoli, & Tallman, 2015). Prior to this period, patients with APL had rapidly fatal courses with a high incidence of bleeding complications. An early study demonstrated that those patients who were untreated or treated with corticosteroids only had a median survival of less than 1 week (Cooperberg & Neiman, 1955). Cytotoxic chemotherapy was initially used, and helped patients achieve a CR, yet provided a low survival rate due to complications and relapses (Huang et al., 2016). The breakthrough in the treatment of APL came with the introduction of ATRA, which increased the remission rate, but approximately 30% of patients would still relapse (Huang et al., 2016). Subsequent trials investigated the combination of ATRA and chemotherapy, which raised the event-free survival rate. Finally, ATO was introduced as an effective agent in the disease process, and several studies have demonstrated its efficacy in combination with both ATRA and anthracycline-based regimens. The largest experience treating this disease process remains with ATRA and anthracycline-based regimens. More recent randomized trials have studied the combination of ATRA plus ATO; results of these trials suggest that these regimens produce at least equivalent outcomes compared with standard regimens of ATRA plus chemotherapy.

## PATHOPHYSIOLOGY

The disease is distinguished by a particular cytogenetic abnormality: a balanced reciprocal translocation of chromosomes 15 and 17. This genetic rearrangement involves the retinoic acid receptor alpha (*RARA*) gene on chromosome 17 and the promyelocytic leukemia gene (*PML*) on chromosome 15. This abnormality is denoted as t(15;17)(q22;q12) (Arber et al., 2016). The *PML* gene is thought to be involved with apoptosis and tumor suppression, and the *RARA* gene is expressed in hematopoietic cells and has a significant role in regulating gene expression related to myeloid differentiation (Melnick & Licht, 1999). The resulting fusion protein prevents the transcription of genes necessary for developing myeloid cells to differentiate beyond the promyelocyte differentiation stage. This results in abnormal promyelocyte accumulation in the blood and bone marrow (Walker & Held-Warmkessel, 2010), which leads to the cytopenias observed upon presentation.

## EPIDEMIOLOGY

Acute promyelocytic leukemia accounts for 5% to 20% of acute myeloid leukemia cases, which translates to 600 to 800 new cases each year in the United States (Yamamoto & Goodman, 2008). The disease affects genders in similar proportions. Some evidence has suggested that there is a higher incidence of APL in Mexico, Central and South America, Italy, and Spain (Matastar, Ritchie, Consedine, Magai, & Neugut, 2006; Yamamoto & Goodman, 2008). Incidence rates for APL were found to be higher among Hispanic children and young adults. Blacks have a lower incidence of acquiring APL than non-Hispanic whites, Hispanics, and Asians (Dores, Devesa, Curtis, Linet, & Morton, 2012). The age distribution of APL patients differs from that for other types of AML in that it affects patients with a similar incidence through young and middle adulthood while relatively sparing elderly patients. Its incidence increases in the second decade of life and reaches a plateau in early adulthood. It then remains constant until it decreases after age 60 (Vickers, Jackson, & Taylor, 2000). The incidence of other subtypes of AML rises in older populations.

## PRESENTATION

Patients with APL can present in a similar manner as those with other subtypes of AML. This can include symptoms related to pancytopenia, such as easy fatigue, weakness, infections, and/or bleeding complications. By the time symptoms develop, the bone marrow of affected patients is often packed with abnormal promyelocytes. Not only does this contribute to blood cell count abnormalities such as leukopenia or leukocytosis, anemia, and thrombocytopenia, this population of malignant cells can provoke a unique, severe coagulopathy with elements of both disseminated intravascular coagulation (DIC) and primary fibrinolysis. This coagulopathy can result in catastrophic bleeding events, including intracranial bleeding. For this reason, APL has a high rate of early mortality and is considered a medical emergency (Park et al., 2011).

## PATHOLOGIC FEATURES

**Morphology**

**Figure 1:**
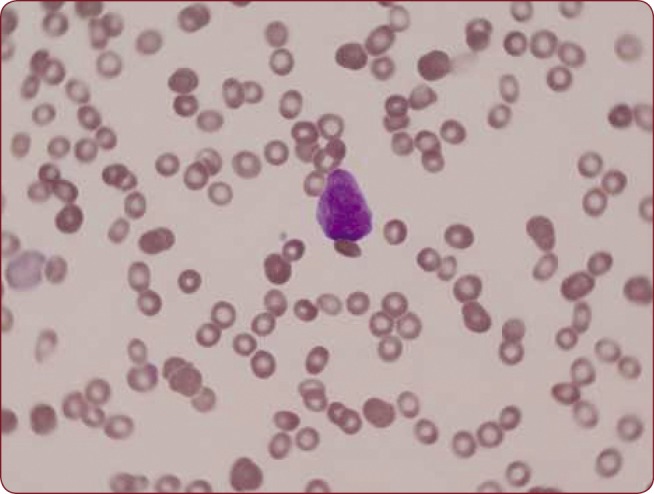
A promyelocyte in a peripheral blood smear in a newly diagnosed APL patient. Image courtesy of Washington University School of Medicine.

**Figure 2:**
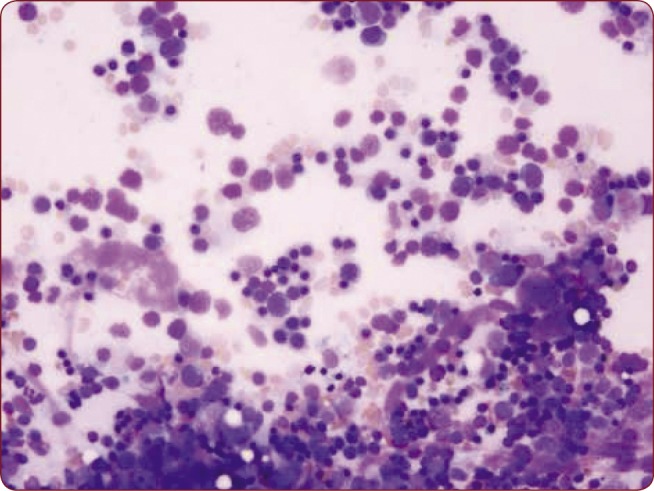
Promyelocytes in a bone marrow aspirate in a newly diagnosed APL patient. Image courtesy of Washington University School of Medicine.

Upon investigation of the bone marrow and peripheral blood of a patient with APL, atypical promyelocytes are seen. Promyelocytes are large (usually greater than 20 microns in diameter) myeloid precursors, often with a high nucleus to cytoplasmic ratio, fine chromatin, and prominent nucleoli. The distinguishing feature of the promyelocyte is the presence of many violet granules in the cytoplasm, which can obscure the nucleus (see Figure 1). The promyelocytes in APL differ from normal promyelocytes in that they are larger in size and have altered shapes of their nuclei (Sainty et al., 2000). The abnormal promyelocyte population in a typical APL sample accounts for at least 30% of observed myeloid cells in the marrow. Very few cells are seen that have progressed past the promyelocyte stage of differentiation (see Figure 2).

The two morphologic variants of the disease are the hypergranular and microgranular forms. The hypergranular form accounts for 75% of cases (Sainty et al., 2000). On Wright’s-stained smears, the cytoplasm of promyelocytes contain tightly packed bright pink, red-blue, or dark purple granules. The microgranular form accounts for the other 25% of APL cases and is characterized by a bilobed nucleus and no granularity visible with light microscopy (McKenna, Parkin, Bloomfield, Sundberg, & Brunning, 1982). Clinicians should be aware of the two variants to improve accuracy of diagnosis and expedite prompt treatment intervention.

**Immunohistochemistry**

With rare exceptions, the APL phenotype is CD34-negative/partial or weak positive, HLA-DR–negative, CD13-positive, CD33-positive, CD11b-negative, CD15-weak or negative, CD117-weak/variable, and sometimes CD2-positive and CD56-positive (Sainty et al., 2000; Sanz et al., 2009).

**Cytogenetics**

Acute promyelocytic leukemia is defined by a particular cytogenetic finding. A reciprocal translocation between the long arms of chromosomes 15 and 17 at q24.1;q21.2 is found, with the subsequent creation of a fusion gene, *PML/RARA*. This genetic abnormality results in the linkage of the *RARA* gene on chromosome 17 with the *PML* gene on chromosome 15 (Melnick & Licht, 1999). The PML/RARa protein functions as an aberrant receptor that blocks retinoic acid–induced myeloid differentiation; hence, the accumulation of promyelocytes arrested in a certain stage of development predominate in affected patients’ tissue samples (Côté et al., 2000). To identify this cytogenetic finding, a variety of testing strategies are routinely employed, including karyotyping, fluorescence in situ hybridization (FISH), and reverse transcription–polymerase chain reaction (RT-PCR) testing.

Karyotyping and FISH are usually used at the time of diagnosis and throughout the treatment course, while RT-PCR testing becomes more relevant later in the treatment course to detect minimal residual disease. Bone marrow specimens are preferred for cytogenetic testing. First, traditional karyotyping is routinely done on diagnostic bone marrow specimens. In this methodology, technicians use programs to analyze a complete karyotype to identify the size, shape, and number of chromosomes. The downside of this method is that it can be time-consuming, taking days to complete. It is, however, highly specific. Fluorescence in situ hybridization should also be utilized in bone marrow specimens, and occasionally in peripheral blood samples when bone marrow sample acquisition is expected to be delayed. In FISH analysis, labeled DNA probes are hybridized to either metaphase chromosomes or interphase nuclei, and the hybridized probes are detected with fluorochromes. This technique is a rapid and sensitive means of detecting recurring numerical and structural abnormalities (Zhang & Le Beau, 2017). The result of the test can usually be available within 24 hours, which is an important advantage, since early diagnosis of this disease subtype is critical.

Finally, RT-PCR testing is another methodology used to detect the *PML/RARA* gene sequences. Reverse transcription–polymerase chain reaction is a highly sensitive technique used for the detection and quantitation of messenger RNA, which consists of two parts: the synthesis of complementary DNA from RNA by reverse transcription and the amplification of a specific complementary DNA by the polymerase chain reaction. This methodology can also detect PML breakpoint location, and can be utilized to monitor minimal residual disease (MRD) status. Currently available PCR methods are able to detect approximately one leukemia cell diluted 10^5^ to 10^6^ times, or one blast or less per 100,000 nucleated cells (Appelbaum et al., 2007). Monitoring of MRD status serially at regular intervals after initial induction therapy can guide treatment choices and identify those patients at higher risk of relapse.

## TREATMENT

**Initial Evaluation**

Upon pathology review of a suspected new acute leukemia patient, any suspicion for APL on initial slide review is an indication to initiate ATRA immediately and urgently (Tallman & Altman, 2009). This should not be delayed until definitive cytogenetic or FISH results are obtained. The addition of ATRA can rapidly improve coagulopathy. If APL is ruled out by cytogenetic testing, ATRA can be discontinued at that time, and appropriate therapy for other types of AML can be initiated (Tallman & Altman, 2009). All-*trans* retinoic acid is a key component in therapy, as it induces differentiation of malignant promyelocytes to neutrophils, which can mitigate the coagulopathy seen in APL patients and potentially prevent hemorrhagic complications, including death.

Patients with diagnosed APL can be divided into one of two categories: low/intermediate risk and high risk. The low/intermediate risk group is defined by an initial white blood cell (WBC) count of less than or equal to 10 × 10^9^/L, while the high-risk group is defined by an initial WBC count of greater than 10 × 10^9^/L. The goal of induction therapy is to reduce the total body leukemia cell population from 10^12^ to below the level of about 10^9^ cells, which is about the level of cytogenetic detection.

**Treatment Regimens**

For low/intermediate risk APL, an accepted induction course is ATRA plus ATO, vs. ATRA plus anthracycline-based chemotherapy. Randomized trials have demonstrated at least the equivalence and in some, the superiority, of ATRA/ATO compared with regimens including ATRA and chemotherapy. The preference for the ATRA/ATO regimen takes into account the lower risk of myelosuppression, cardiac toxicity, side effects common with anthracycline-based regimens, and risk of secondary leukemias (Lallemand-Breitenbach & de Thé, 2013). The Intergroup APL0406 randomized phase III trial demonstrated similar rates of CR, fewer deaths during induction, and superior 2- and 4-year event-free survival and overall survival with ATRA/ATO compared with ATRA plus idarubicin (Lo-Coco et al., 2013). Induction therapy consisted of ATO at 0.15 mg/kg/day and ATRA at 45 mg/m^2^/day until a CR was confirmed. At that time, patients randomized to the ATRA/ATO arm went on to receive a consolidation regimen consisting of ATO at 0.15 mg/kg/day 5 days/wk from weeks 1–4, 9–12, 17–20, 25–28, and ATRA at 45 mg/m²/day for 15 days at a time from weeks 1–2, 5–6, 9–10, 13–14, 17–18, 21–22, and 25–26 (Lo-Coco et al., 2013; Table 1). When compared with those patients who received ATRA plus anthracycline-based regimens, the event-free survival rates were 97% in the ATRA/ATO group vs. 85% in the ATRA/chemotherapy group (Lo-Coco et al., 2013).

**Table 1:**
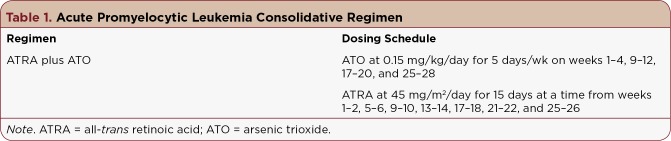
Acute Promyelocytic Leukemia Consolidative Regimen

As many studies investigating the efficacy of ATRA/ATO-based regimens involved only patients in the low/intermediate risk group, an appropriate regimen to treat those patients who fall into a high-risk category remains less clear. The current standard of care for these patients continues to be ATRA in combination with anthracycline-based regimens, although studies are currently underway to investigate the possibility of using ATRA/ATO as an effective treatment regimen for this group. This combination is supported by data that revealed that patients treated with ATRA in combination with chemotherapy had superior rates of CR and disease-free survival when compared with patients who received chemotherapy alone (Fenaux et al., 1993; Tallman et al., 1997). Choices for chemotherapy regimens include anthracycline-based regimens, due to the susceptibility of APL cells to this drug class, due to the low expression of the drug efflux pump P-glycoprotein on their cell membrane (Candoni et al., 2003). Since no direct comparisons have been done comparing anthracycline agents, regimen choices can vary. Randomized trials used daunorubicin, cytarabine, and ATRA for induction therapy and reported CR rates of 80% to 95% (Burnett, Grimwade, Solomon, Wheatley, & Goldstone, 1999; Fenaux et al., 1993, 1999; Tallman et al., 2002; see Table 2).

**Table 2:**
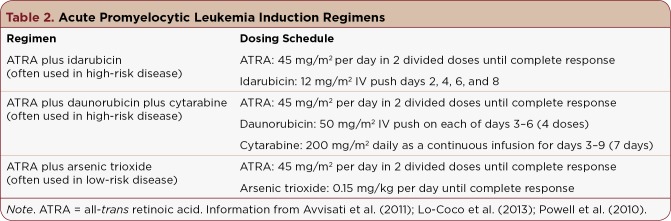
Acute Promyelocytic Leukemia Induction Regimens

Another retrospective analysis studied patients treated with ATRA plus idarubicin induction and reported a CR rate of 91% (de la Serna et al., 2008). Furthermore, the Australian APML4 trial treated patients with idarubicin, ATRA, and ATO, in which 95% of patients achieved a hematologic CR, and following 2 cycles of consolidation with ATRA and ATO, all patients achieved a molecular CR and received 2 years of maintenance therapy with ATRA, oral methotrexate (MTX), and 6-mercaptopurine (6-MP). Disease-free survival at 2 years was 98% (Iland et al., 2012).

Clinicians treating this subtype of leukemia need to be aware of how patients can be expected to respond when treated with various regimens. With ATO/ATRA regimens, a rise in WBC count can occur with treatment, and this rising white count can also coincide with the risk of differentiation syndrome, which will be discussed in greater detail in a following section. The rising count may need to be controlled with hydroxyurea. In contrast, regimens with combined ATRA and chemotherapy can lead to a rapid pancytopenia after the initiation of treatment.

**Maintenance Therapy**

For low- to intermediate-risk patients who achieve a complete molecular remission with regimens including ATRA and ATO, maintenance therapy is not routinely recommended, as benefits are as of yet unproven and the risk of toxicity increases (Coutre et al., 2014; Lo-Coco et al., 2013). For high-risk populations, maintenance is recommended due to the potential for a disease-free survival benefit. This is based on early randomized trials of ATRA plus chemotherapy in which, when compared with observation, patients assigned to ATRA maintenance demonstrated superior rates of disease-free survival at 5 years and a lower 10-year cumulative incidence of relapse (Adàs et al., 2010; Tallman et al., 2002). These results have led to the frequent use of maintenance therapy in all APL patients, although it may not be necessary. Further trials are needed to answer this clinical question.

When utilized, standard maintenance regimens include single-agent ATRA at 45 mg/m² po for 7 days repeated every other week for 1 year or combination regimens such as ATRA at 45 mg/m² po daily on an intermittent schedule (15 days every 3 months or 7 days every 2 weeks) plus 6-MP 60 mg/m² po every evening plus MTX 20 mg/m²/wk as tolerated. In the combination regimen, the medications are taken for 1 year and adjusted as needed for myelosuppression or liver function abnormalities (Powell et al., 2010, 2011).

## EVALUATING FOR RESPONSE

**Induction**

The goal of induction therapy is the attainment of a morphologic CR with recovery of normal hematopoiesis. Molecular remission can be obtained after induction therapy, but can also occur after consolidation therapy. Response to induction therapy can be obtained with a bone marrow biopsy when patients recover an absolute neutrophil count of greater than 1,000/μL and platelet count of greater than 100,000/μL, and no longer require red cell transfusions, which usually occurs between days 30 to 35 from the start of induction therapy. An earlier bone marrow biopsy, such as a day 14 sample, is not recommended, as promyelocytes can persist in the marrow at this time and be misleading (Sanz et al., 2009). For those patients who achieve a morphologic remission, proceeding directly to consolidative therapy is indicated.

**Consolidation**

Patients should be evaluated after the completion of consolidation with a bone marrow biopsy and aspiration. The sample should be sent for the PML/RARa fusion transcript using RT-PCR. The goal of APL consolidation is to achieve a molecular CR (CRm), as defined by a negative RT-PCR test (Cheson et al., 2003). If a positive RT-PCR test is noted after consolidation, the test should be repeated in 4 weeks. If negative at that time, the patient can proceed to maintenance therapy. If the test remains positive, the patient will need treatment for refractory disease.

## UNIQUE COMPLICATIONS DURING THERAPY

**Disseminated Intravascular Coagulation**

A unique and potentially devastating complication of APL is a coagulopathy resulting from DIC and primary fibrinolysis. This can be noted upon presentation or during therapy. Patients often have hypofibrinogenemia, elevated prothrombin time/partial thromboplastin time, and thrombocytopenia. Coagulation parameters should be monitored closely, especially early in the course, and replenished aggressively. Common transfusion goals include a platelet threshold of 20,000 to 30,000/μL, fibrinogen levels above 150 mg/dL, and international normalized ratio levels below 2 with infusion of platelets, cryoprecipitate, and fresh frozen plasma, respectively (Sanz, Tallman, & Lo-Coco, 2005; Tallman & Altman, 2009; Table 3). Invasive procedures should be avoided where possible until coagulopathy is controlled.

**Table 3:**
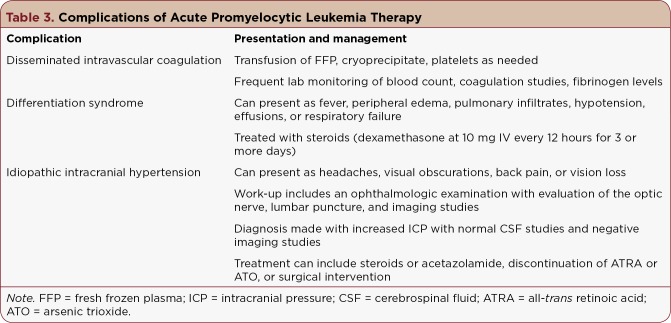
Complications of Acute Promyelocytic Leukemia Therapy

**Differentiation Syndrome**

The differentiation syndrome is another unique complication seen during APL treatment. This has also been described as retinoid acid syndrome or cytokine storm. The syndrome occurs in about 25% of patients within 2 to 21 days after initiation of treatment and is seen more often in those patients with a higher WBC count at diagnosis (Montesinos et al., 2009; Vahdat et al., 1994). Its presentation can be greatly varied and can include fever, peripheral edema, pulmonary infiltrates, respiratory failure, hypotension, fluid accumulations including pleural or pericardial effusions, or renal and hepatic dysfunction. It can be accompanied by hyperleukocytosis. While the pathogenesis of differentiation syndrome is not completely clear, it has been speculated that ATRA or ATO causes a cascade of events leading to a systemic inflammatory response syndrome, which includes endothelium damage with capillary leak syndrome, occlusion of microcirculation, and tissue infiltration. This inflammatory response is thought to be mediated by differentiating myeloid cells, such as interleukin-1 (IL-1), interleukin beta (IL-beta), interleukin-6 (IL-6), and interleukin-8 (IL-8; Luesink et al., 2009). Another hypothesis is that ATRA induces changes in the adhesive properties of APL cells that promote the aggregation of promyelocytes, leukostasis, and vessel occlusion, as well as leukocyte migration from the blood into the tissue (Che-Pin, Huang, Chang, Lin, & Sheu, 2000).

Primary treatment includes aggressive management with steroids (usually dexamethasone at 10 mg IV every 12 hours for 3 or more days; Sanz et al., 2009). Without treatment with glucocorticoids, patients who develop a differentiation syndrome have a 30% mortality rate, usually from catastrophic respiratory failure or neurologic events. With treatment, patients typically respond within 12 hours with symptom improvement, and mortality rate drops to about 5% (Tallman et al., 2000). A high level of suspicion for differentiation syndrome when clinical changes occur is necessary, as prompt initiation of treatment is imperative. The role of prophylactic glucocorticoids has been questioned, but not formally studied in a large randomized trial. Analysis of other trials that have used prophylactic steroids exhibited similar rates of differentiation syndrome, so it is not a common practice to add this element to regimens. Despite data to prove prophylaxis as effective in preventing the syndrome, some experts will add prophylactic steroids in particular cases, such as in those patients with renal dysfunction or higher WBC counts (Kelaidi et al., 2009; Sanz & Montesinos, 2014). Stopping ATRA or ATO therapy is often not indicated and does not improve outcomes in more stable patients (Tallman et al., 2000). For patients who develop multisystem organ failure or require intensive care admission for respiratory failure, ATRA and/or ATO are often discontinued until symptoms completely resolve, although no clear randomized studies demonstrate this as a clear indication to hold these agents.

Supportive care is a key element in the care of affected patients. Respiratory failure can indicate the need for positive pressure ventilation, including intubation. Hemodynamic compromise can lead to the need for vasopressor support or volume resuscitation with blood products and intravenous fluids. As these patients are immunosuppressed, an aggressive infectious work-up is indicated in the setting of clinical deterioration, with the addition of broad-spectrum antibiotics.

**Idiopathic Intracranial Hypertension**

Rarely, idiopathic intracranial hypertension can affect patients undergoing treatment with ATRA. The condition affects children and adolescents at a higher frequency than adults (Sanz et al., 2009). Idiopathic intracranial hypertension is suspected in patients reporting headaches, visual obscurations, back pain, or vision loss (Wall et al., 2014). Work-up includes an ophthalmologic examination with evaluation of the optic nerve, lumbar puncture, and imaging studies. The diagnosis can be made with the finding of increased intracranial pressure with normal cerebrospinal fluids and negative imaging studies, such as computed tomography or magnetic resonance imaging scans. Papilledema is a common finding, but not necessary for diagnosis. Treatment can include temporary discontinuation or dose reduction of ATRA, the administration of steroids or acetazolamide, or surgical intervention (Corbett & Thompson, 1989; Matthews, 2008).

## SUMMARY

Acute promyelocytic leukemia is a unique, distinct disease process that requires a high level of clinical suspicion and urgent initiation of therapy. A thorough understanding of the presentation and pathophysiology can help to guide the advanced practitioner to identify affected patients and improve clinical care. The incorporation of ATRA and ATO into treatment regimens has led to excellent remission rates and long-term survival. However, patients remain at risk for early death due to complications like bleeding and differentiation syndrome. Induction regimens are varied and are chosen based on risk category upon presentation. Further trials are needed to elucidate the role of nonchemotherapy induction regimens for high-risk patients. Furthermore, unique treatment complications can arise, and the advanced practitioner, along with the inpatient staff, is integral in identifying these potential events and intervening promptly.
